# Immune pathways and prenatal/perinatal environmental exposures contribute to epigenetic gestational age prediction and acceleration

**DOI:** 10.1080/15592294.2025.2610521

**Published:** 2026-01-20

**Authors:** Amy A. Eapen, Ian M. Loveless, Mingming Pan, Xiaoyu Liang, Audrey Urquhart, Jennifer K. Straughen, Andrea E. Cassidy-Bushrow, Alexandra R. Sitarik, Neil Simmerman, Emma Thompson, Leah Kottyan, Carole Ober, Christine C. Johnson, Edward Zoratti, Albert M. Levin

**Affiliations:** a Division of Allergy and Immunology, Department of Internal Medicine, Henry Ford Health + Michigan State University Health Sciences, Detroit, MI, USA; b Department of Medicine, College of Human Medicine, Michigan State University, East Lansing, MI, USA; c Center for Bioinformatics, Henry Ford Health + Michigan State University Health Sciences, Detroit, MI, USA; d Department of Public Health Sciences, Henry Ford Health + Michigan State University Health Sciences, Detroit, MI, USA; e Department of Epidemiology and Biostatistics, Michigan State University, East Lansing, MI, USA; f Department of Obstetrics, Gynecology and Reproductive Biology, College of Human Medicine, Michigan State University, East Lansing, MI, USA; g Department of Pediatrics and Human Development, College of Human Medicine, Michigan State University, East Lansing, MI, USA; h Department of Women’s Health Services, Henry Ford Health + Michigan State University Health Sciences, Detroit, MI, USA; i Department of Human Genetics, University of Chicago, Chicago, IL, USA; j Center of Autoimmune Genomics and Etiology, Division of Eosinophilic Disorders, Cincinnati Children’s Hospital Medical Center, Cincinnati, OH, USA

**Keywords:** Gestational age, epigenetic gestational age clock, prenatal environment

## Abstract

DNA methylation (DNAm), capturing chronological gestational age (GA) and epigenetic gestational age acceleration (EGAA), can be modified by environmental exposures. The Asthma&Allergy array is a new DNAm array developed with content focused on asthma and allergy loci. The association between content on the Asthma&Allergy array and chronological GA and EGAA has not been evaluated alone or in the context of prenatal/perinatal exposures. We performed an epigenome wide association study (EWAS) chronological GA at single CpG sites and regions in cord blood from 391 newborn children from a Detroit-based birth cohort. We further constructed a multi-CpG site methylation model to predict chronological GA. Also, associations between prenatal/perinatal environmental factors with GA, epigenetic gestational age (EGA), and EGAA were assessed. We identified 2,435 CpG sites associated with chronological GA, and CpGs within the HLA class II locus (*HLA-DRB1, HLA-DQB1, HLA-DRB6*) were among the most significantly associated with chronological GA. Our multi-CpG site model attained higher predictive accuracy (R^2^ = 0.88) comparable to other published methods. Using genes implicated in region-based analyses (*n* = 395 regions), the pathways most significantly enriched with chronological GA-associated CpGs included T helper 1(Th1) and 2(Th2) activation, B-cell development, and IL-10 signaling, which were also enriched in at least one of the other published epigenetic GA clocks. In multi-exposure models, infant’s first-born status and maternal parity were associated with EGAA. Our findings highlight enrichment for T cell modulated pathways and antigen presentation as biological processes associated with chronological GA, as well as prenatal/perinatal factors that may affect EGAA.

## Introduction

Chronological gestational age (GA) is associated with developmental maturity, and both pre- and post-term births are associated with risk of adverse outcomes in both early childhood and also later on in life [1–3]. However, clinically measured chronological GA (by last menstrual period or ultrasound) is only one estimation of GA. Biological GA, the physiological state of cells within tissues, is another estimate of GA and is influenced by genetic and environmental factors [4]. DNA methylation (DNAm), which is influenced by the environment and genetics, has been utilized to estimate GA and create epigenetic gestational age (EGA) clocks [5]. The discordance between one’s epigenetic age and chronological age has been proposed as an estimate of whether individuals are biologically aging faster or slower relative to their chronological age [6], which has been termed epigenetic age acceleration, or epigenetic gestational age acceleration (EGAA) when examining GA. EGAA has been associated with risk of a variety of childhood-onset conditions including, but not limited to, developmental delay and cardiometabolic disorders [7,8].

Existing DNAm-based EGA clocks have generally utilized CpG sites included on Illumina methylation arrays (HumanMethylation 27 K, 450 K, and HumanMethylationEPIC 850K (EPIC)). While these arrays provide differing degrees of epigenome-wide coverage and have been successful at developing accurate GA clocks, they comprise <5% of the CpG sites across the human epigenome. As such, they have not comprehensively probed the contribution of DNAm to GA in loci that are associated with conditions that are impacted by biological pathways in aging. Specifically, childhood asthma and allergy has been associated with EGAA [9], but commercial high-throughput arrays have limited representation of immunological pathways involved in these diseases [10].

Recently, leveraging whole genome bisulfite sequencing (WGBS) and *in silico* evidence of gene regulatory regions [11], Morin developed a custom-content DNAm array [10] that includes enhanced coverage of CpGs overlapping with predicted enhancers and transcription factor binding sites within loci associated with asthma and allergic disorders that complements the Illumina EPIC array, and this custom-content array is commonly referred to as the Asthma&Allergy array. Specifically, it covers CpGs that are not present on the Illumina EPIC array and encompasses areas of the genome that are enriched for immune-related diseases. As biological aging has shown to be associated with risk of these conditions and immune pathways [12], it would be enlightening to characterize the relationship between DNAm captured on this array and GA [13].

In the current study, we applied the Asthma&Allergy array to cord blood DNA from a large metro-Detroit birth cohort that is diverse in terms of socioeconomic status (SES), parental-reported race, and urban/suburban residence to investigate the association between DNAm and chronological GA. We first identified individual CpG sites and regions of DNAm associated with GA and assessed their predicted biological pathway enrichment. Second, we developed a DNAm GA clock from the Asthma&Allergy array and evaluated its accuracy in predicting chronological GA. Finally, as the environment (e.g., environmental tobacco smoke exposure (ETS), pet exposure, pollutant exposure, stress) [14] can modify DNAm, we also investigated the associations of prenatal and perinatal environmental factors with EGA and EGAA in both individual and multi-exposure models.

## Materials and methods

### Study cohort

The Wayne County Health, Environment, Allergy and Asthma Longitudinal Study (WHEALS) is a birth cohort from southeastern Michigan that has previously been described [15,16]. Briefly, women were eligible for inclusion in the study if they were in their second or third trimester of pregnancy, were between 21 and 49 years of age, and lived in a predefined cluster of zip codes in Detroit and its surrounding Wayne County suburbs. A total of 1258 pregnant women were enrolled from September 2003 to December 2007 with no exclusion based on GA. Informed written consent was obtained for cord blood collection, as well as the use of DNA for future assays. Mothers had the option to opt out of the genetic portion if desired and still remain in the study. This research was approved by the Henry Ford Health Institutional Review Board, protocol approval number 14914 and 1881–29 (genetics sub-consent approved September 28, 2004), and adhered to the Declaration of Helsinki guidelines.

Cord blood was collected at delivery, and genomic DNA was isolated from whole cord blood using the QIAGEN FlexiGene DNA Kit (Germantown, MD). Of the 1,258 recruited women, 763 children (76.1%) either completed a 2-year follow-up visit in the clinic or had blood drawn for measurement of immunoglobulin E (primary outcome of parent study). From this subset who also had cord DNA stored, we randomly selected 391 participants for DNAm assessment using the Asthma&Allergy array. Chronological gestational age was chart abstracted from the participant’s obstetrician’s calculation in the electronic medical record, either ultrasound-based or from last menstrual period.

### DNAm quality control and quantification

Raw methylation Illumina *idat* files from the 391 cord DNA Asthma&Allergy array were loaded into R version 4.2.1 using the *minfi* package [17]. A total of 45,954 CpG sites were included on the array before quality control (QC). Probes with detection p-values greater than 0.05, with single nucleotide polymorphisms SNPs at either the 3’ or 5’ locations, on the sex chromosomes, or capturing CpG sites not intended to be targeted by the array were removed. After these exclusions, 45,296 CpG sites remained for analysis, and the resulting data were quantile normalized using the *ENmix* package in R [18]. Lastly, the resultant CpG site beta values (i.e., estimated proportion of methylation) were converted to logit transformed M-values, which were used for subsequent analyses. Based on visual inspection of CpG site beta-value distribution curves and epigenome-wide principal component plots, we failed to identify any outliers and maintained all samples for downstream analysis.

### Statistical methods

Descriptive statistics of demographic characteristics and prenatal/perinatal environmental exposures are summarized in Table 1. We used mean and standard deviation to summarize quantitative measures and counts and percentages for categorical measures. Univariate associations between each one of these factors and chronological GA was calculated using linear regression, with an F-test used to assess statistical significance.

**Table 1.** Demographic characteristics of WHEALS participants stratified by cord DNA methylation data availability.

Cord blood DNA methylation data availability							
			level	Overall	No	Yes	*p*-value*
	n			1258	867	391	
Demographic	Mom Race (%)		African American	778 (61.8)	539 (62.2)	239 (61.1)	0.766
			European American	290 (23.1)	195 (22.5)	95 (24.3)	
			Other	190 (15.1)	133 (15.3)	57 (14.6)	
	Maternal Education (%)		≤HS diploma	74 (5.9)	58 (6.7)	16 (4.1)	4.40 × 10^−4^
			HS diploma	228 (18.1)	173 (20.0)	55 (14.1)	
			Some college	605 (48.1)	421 (48.6)	184 (47.1)	
			Bachelor’s degree	351 (27.9)	215 (24.8)	136 (34.8)	
	Marital Status (%)		Not Married	485 (38.6)	355 (40.9)	130 (33.2)	0.011
			Married	773 (61.4)	512 (59.1)	261 (66.8)	
	Total Household Income (%)		<20k	182 (14.5)	130 (15.0)	52 (13.3)	0.290
			20k- <40k	295 (23.4)	214 (24.7)	81 (20.7)	
			40k- <80k	347 (27.6)	240 (27.7)	107 (27.4)	
			80k- <100k	135 (10.7)	85 (9.8)	50 (12.8)	
			≥100k	148 (11.8)	95 (11.0)	53 (13.6)	
			refuse to answer	151 (12.0)	103 (11.9)	48 (12.3)	
	Urban Residence (%)		Suburban	555 (44.1)	385 (44.4)	170 (43.5)	0.806
			Urban (Detroit)	703 (55.9)	482 (55.6)	221 (56.5)	
	Maternal Age (mean (SD))			29.56 (5.24)	29.37 (5.21)	29.98 (5.30)	0.056
	Paternal Age (mean (SD))			32.27 (6.78)	32.14 (6.74)	32.57 (6.86)	0.313
Pregnancy and Delivery	Infant’s Sex (%)		male	622 (49.5)	421 (48.6)	201 (51.4)	0.392
			female	635 (50.5)	445 (51.4)	190 (48.6)	
	Infant’s Race (%)		African American	780 (62.0)	536 (61.8)	244 (62.4)	0.491
			European American	290 (23.1)	195 (22.5)	95 (24.3)	
			Other	188 (14.9)	136 (15.7)	52 (13.3)	
	Chronological Gestational Age Days (mean (SD))			272.61 (12.21)	271.95 (12.67)	274.02 (11.02)	0.005
	Parity (mean (SD))			1.14 (1.23)	1.16 (1.28)	1.09 (1.13)	0.388
		BMI First Recorded in Pregnancy (mean (SD))		30.66 (8.25)	30.76 (8.19)	30.51 (8.36)	0.639
		BMI Last Recorded in Pregnancy (mean (SD))		35.08 (7.77)	35.17 (7.69)	34.95 (7.89)	0.678
		Birth Weight z-score (mean (SD))		−0.12 (0.99)	−0.17 (0.99)	−0.01 (0.98)	0.007
		Delivery type (%)	Vaginal	791 (63.2)	542 (63.0)	249 (63.7)	0.853
			C-section	461 (36.8)	319 (37.0)	142 (36.3)	
		Mode of Delivery (type) (%)	vaginal	791 (64.0)	542 (64.1)	249 (63.8)	0.282
			Planned C-section	209 (16.9)	135 (16.0)	74 (19.0)	
			Unplanned C-section	236 (19.1)	169 (20.0)	67 (17.2)	
		First Born (%)	No	798 (63.4)	543 (62.6)	255 (65.2)	0.413
			Yes	460 (36.6)	324 (37.4)	136 (34.8)	
		Number of Previous Pregnancies	0%)	286 (22.7)	196 (22.6)	90 (23.0)	0.073
			1	292 (23.2)	200 (23.1)	92 (23.5)	
			2	269 (21.4)	170 (19.6)	99 (25.3)	
			3	186 (14.8)	132 (15.2)	54 (13.8)	
			≥4	225 (17.9)	169 (19.5)	56 (14.3)	
		Maternal Antibiotic Use During	No Pregnancy (%)	399 (44.8)	230 (44.4)	169 (45.4)	0.813
			Yes	491 (55.2)	288 (55.6)	203 (54.6)	
		Maternal Antifungal Use During	No Pregnancy (%)	725 (81.5)	419 (80.9)	306 (82.3)	0.666
			Yes	165 (18.5)	99 (19.1)	66 (17.7)	
		Born Season (%)	Winter	257 (20.4)	175 (20.2)	82 (21.0)	0.907
			Spring	285 (22.7)	200 (23.1)	85 (21.7)	
			Summer	345 (27.4)	234 (27.0)	111 (28.4)	
			Fall	371 (29.5)	258 (29.8)	113 (28.9)	
		Mom Smoking Status (%)	No	1108 (88.1)	761 (87.8)	347 (88.7)	0.690
			Yes	150 (11.9)	106 (12.2)	44 (11.3)	
Environmental Exposure During Pregnancy		Environmental Tobacco Smoke	No (%)	911 (72.4)	619 (71.4)	292 (74.7)	0.255
			level	Overall	No	Yes	P-value*
			Yes	347 (27.6)	248 (28.6)	99 (25.3)	
Indoor Pet(s) (%)			No pet(s)	820 (65.2)	573 (66.1)	247 (63.2)	0.346
			Pets	438 (34.8)	294 (33.9)	144 (36.8)	
Outdoor Pets (%)			No pets	1214 (96.5)	839 (96.8)	375 (95.9)	0.545
			Pets	44 (3.5)	28 (3.2)	16 (4.1)	

Abbreviations: %, column percentage; BMI, body mass index; C-section, caesarean section; k, thousand; HS, high school; N, count; SD, standard deviation; WHEALS, Wayne County Health, Environment, Allergy and Asthma Longitudinal study.

*Chi-square and t-test *p*-values evaluating significant differences for respective categorical and continuous variables between WHEALS participants with and without cord blood DNA methylation data.

Three primary analyses were used to evaluate the association between DNAm and chronological GA. First, an epigenome-wide association study (EWAS) was performed to identify CpG sites associated with chronological GA (in days) using linear regression, adjusting for infant’s sex, parental-reported infant race, and latent features identified using the CorrConf package [18]. These latent features were identified treating chronological GA as the outcome and adjusting for infant’s sex and race. Association p-values were corrected for multiple-testing using a Benjamini and Hochberg false discovery rate (FDR) threshold of 0.05 [19]. Second, a region-based assessment was conducted where differentially methylated regions (DMRs) were identified based on combinations of p-values from the single site EWAS using *comb-p* [20], which was implemented in the Enmix R package [21]. Individual CpG sites from both the single site EWAS and within DMRs were then functionally annotated using the ChiPseeker R package [22]. To assess whether these genes were expressed in cord blood, we compared the annotated genes to cord blood RNA-Seq datasets from the Gene Expression Omnibus (GEO). We further tested for enrichment of FDR-significant CpG site functional location (promoter, exon, intron, intergenic, 5` UTR, 3` UTR, and downstream) using a hypergeometric test, to identify locations found in excess or in deficit relative to their respective proportions on the whole Asthma&Allergy array. Following nearest gene assignment of CpGs within FDR-significant regions, canonical pathway analyses were performed using Ingenuity Pathways Analysis (IPA) [23]. To account for the gene coverage on the array, the Fisher’s exact enrichment tests were performed using all genes functionally annotated to CpG sites on the array as a background (i.e., as opposed to the standard analysis which uses all annotated genes in the genome).

Third, a multi-CpG site model of chronological GA was constructed using elastic net penalized linear regression, and Supplementary Figure S1 visually depicts our modelling procedure. For model construction and validation, the cohort was divided into training (*n* = 313, 80%) and testing (*n* = 78, 20%) sets. As input to the model, CpG sites that met an FDR-corrected p-value threshold of 0.05 from the training set were included, and following this feature selection step, the subsequent penalized model was adjusted for infant’s sex, parent-reported race of the infant, and latent factors calculated using CorrConf [18]. Within the training data, EGA estimates were calculated for each sample using five-fold cross-validation. In this process, one sample was identified that had a residual greater than 20 times the mean. This sample was determined to be an outlier and removed from the model construction training set, resulting in a total training sample size of 312. Next, the final model was applied to the testing dataset to assess its ability to estimate chronological GA using Pearson’s correlation, the intraclass correlation coefficient (ICC), and the percentage of variation explained (ie. linear regression R^2^). A Bland-Altman plot was also used to visualize the agreement between chronological GA and EGA across chronological GA [24].

Finally, the association between prenatal/perinatal environmental factors (obtained by questionnaire) and GA measures were assessed using linear regression. For these analyses, GA was measured in three ways: 1) chronological GA, 2) EGA, and 3) EGAA. To obtain unbiased estimates of EGA and EGAA for each individual in the entire cohort, we used a five-fold cross-validation of the approach described in Supplementary Figure S1. Briefly, each sample from the entire cohort (*n* = 390) was apportioned into one of five folds. In each round, 4/5 of the data was used to construct the model (ie, EWAS CpG sites selection followed by penalized model construction), which was subsequently used to predict the chronological GA of the samples from the fold left out. Repeating this procedure over the five rounds, each individual is assigned a predicted EGA estimate. EGAA was estimated as the difference between the EGA and the observed chronological GA (i.e., the residuals), such that a positive value would be consistent with biological age acceleration in days. A series of 22 unadjusted, single prenatal/perinatal environmental exposure models were fit for each GA type. To account for the role of multiple environmental factors affecting GA measures, a multi-exposure model was constructed separately for each of the GA measures using a backward selection approach. In each multi-exposure model, the model construction began with the inclusion of all 22 perinatal factors, and the Akaike Information Criteria (AIC) was subsequently utilized to determine those environmental exposures that were retained. The selection was performed using the ‘step’ function in R 4.2.1. statistical programming language [25].

## Results

### Cohort characteristics

As previously described, WHEALS represents a diverse sampling of the population in the metro-Detroit area [15,16]. The sub-cohort of WHEALS in this study included 391 individuals, and demographic and prenatal/perinatal characteristics for this sample are described in Table 1. Characteristics of our sub-cohort were similar to the full WHEALS cohort, with the exceptions that our sub-cohort included more married mothers (*p* = 0.011), higher maternal education (*p* = 4.40×10^−4^), higher chronological GA (*p* = 0.005), and higher birth weight Z-score (*p* = 0.007). In terms of race, 63% of infant participants were African American (*n* = 244), 24% European American (*n* = 95), and 13% other (*n* = 52), which included Hispanic, Arabic, and individuals reporting mixed race. The average maternal age at birth was 29.98 y (standard deviation (SD) = 5.3), and 57% (*n* = 221) of participants lived in an urban setting (defined as having a home in a Detroit-city limit ZIP code), while the remaining 44% lived in suburban settings (ZIP code outside of Detroit). 51% (*n* = 201) of infants were male, and 64% (*n* = 249) of infants were born by vaginal delivery.

### Single CpG site and region-based DNAm associations with chronological GA

After correction for multiple tests, 2,435 CpGs were associated (FDR-adjusted *p*-value < 0.05) with chronological GA. A quantile-quantile plot of the single CpG site p-values (Supplementary Figure S2) showed no inflation (genome-wide inflation = 0.998), and the results for all single CpG site analyses are presented in Supplementary Figure S1. For 1,330 (55%) of these CpG sites, increasing methylation was associated with increasing GA, and for the remaining 1,105 (45%) CpG sites, increasing methylation was associated with decreasing GA. Overall, 992 unique genes were annotated to these CpGs. To assess whether these genes were expressed in cord blood, we compared the 992 annotated genes to a cord blood RNA-seq dataset from GEO and noted that they are all expressed [26]. The majority of the top significant CpGs associated with increased GA were located within chromosome 6, with the CpGs annotated to histone genes *H2BC10 and H2BC13*. In comparison, most CpGs associated with decreased GA were spread across different chromosomes.

Next, the CpG sites associated with chronological GA were classified into functional annotation categories within the genome, and the percentages falling into each of these categories are included in Table 2, accompanied by the respective percentages for all of the CpGs included on the array. Comparing the values for each category, there is evidence of deviation from a random selection of CpGs associated with chronological GA, with functional annotation categories both significantly over- and under-represented. Specifically, compared to the distribution of all CpGs on the array, enrichment of GA-associated CpGs was observed within the first introns (*p* = 3.83×10^−18^) and other introns (*p* = 1.14×10^−9^), 3’ untranslated regions (*p* = 0.016), and promoters located 1–2 kb upstream of the transcription start site (*p* = 0.004). In contrast, there was a significant deficit of GA-associated CpGs within the first 1kb of promoters (*p* = 2.04×10^−48^).

**Table 2.** Functional annotation distribution of CpG sites associated with chronological gestational age. Observed % is the percentage of significant GA associated CpGs that fall into each functional annotation category. The expected % is the percentage of CpG on the Asthma&Allergy array that fall into each annotation category irrespective of association with GA. Underrepresentation is testing whether we observed fewer than expected GA-associated CpG in a given annotation category, and overrepresentation is testing whether we observed more than expected GA-associated CpG in a given annotation category.

			*p*-value*	
Functional annotation categories	Observed %	Expected %	Underrepresentation	Overrepresentation
Promoter (≤1kb)	16.3	28.7	2.04x10^−48^	1
Promoter (1-2kb)	12.7	11	0.997	0.004
Promoter (2-3kb)	4.9	5.2	0.271	0.760
5’ UTR	0.1	0.2	0.142	0.954
3’ UTR	2.7	2.1	0.989	0.016
1st Exon	0.5	0.5	0.460	0.658
Other Exon	1.7	2.3	0.019	0.987
1st Intron	17.3	11.5	1	3.83x10^−18^
Other Intron	24.5	19.7	1	1.14x10^−09^
Downstream (≤300)	0.4	0.2	0.977	0.053

*P-values were generated using a hypergeometric test to identify functional annotation categories found in excess or in deficit relative to the respective proportions on the whole array.

**Figure 1.**
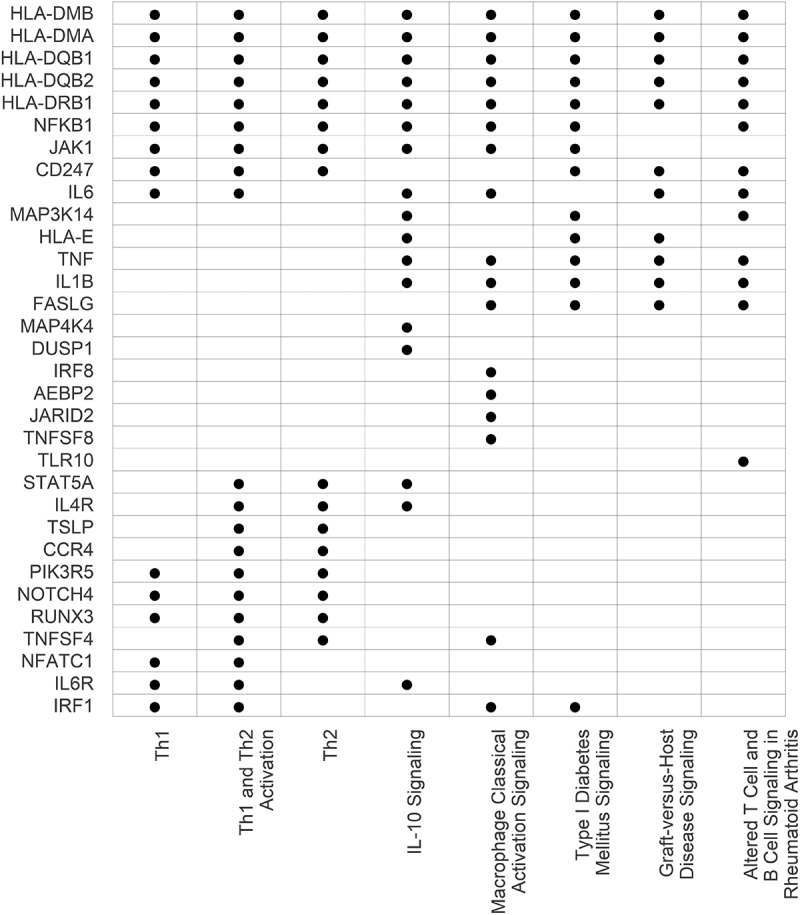
Genes included across top enriched pathways for for chronological gestational age based on CpG region analyses. From the single CpG sites epigenome wide association study with chronological age, we identified differentially methylated regions based on combinations of p-values from the single site EWAS using *comb-p*. These regions were then mapped to genes by ChiPseeker. Pathway analyses using IPA were performed. The table lists the pathways with an enrichment *p*-value < 1x10^−5^. Dot represents inclusion of gene in each listed pathway.

In addition to the single CpG site associations, a region-based analysis was conducted using the *comb-p* approach [20]. In this analysis, 395 regions were significantly associated with GA (FDR adjusted *p*-values < 0.05) (Supplementary Table S2). These regions were comprised of 2066 CpG sites which were annotated to 336 genes. Of these genes identified by the region-based analysis, 329 (97.9%) overlapped with the 992 identified via the single CpG site analysis, with the following seven genes uniquely identified by the region-based approach: *PRSS16, EID3, MIR4706, SCARF2, FBXW2, IKZF5, LYSMD1*. All annotated genes implicated in the region-based analyses were then assessed for canonical biologic pathway enrichment using IPA, with the list of unique genes annotated to CpGs covered on the array as the background. This analysis revealed that the genes nearest to CpGs associated with chronological GA were significantly enriched in pathways related to immune function. Specifically, the top eight most significant pathways (with cutoff *p* < 1x10^−5^) are listed in Figure 1, and all enriched pathways (*p* < 0.05) are listed in Supplementary Table S3. Enriched pathways included T helper 1 (Th1) and 2 (Th2) activation, macrophage classical activation signaling, and IL-10 signaling. Genes that overlapped with all of the top eight pathways included MHC class II, specifically *HLA-DMA*, *HLA-DMB, HLA-DQB1*, *HLA-DQB2*, and *HLA-DRB1*. *NFKB1* was enriched in seven out of the top eight pathways while *CD247*, *IL-6*, and *JAK* were enriched in six out of the top eight. *IL1B* was included in IL-10 signaling and macrophage classical activation signaling, with absence in the *T*- cell enriched pathways.

### EGA clock

**Figure 2.**
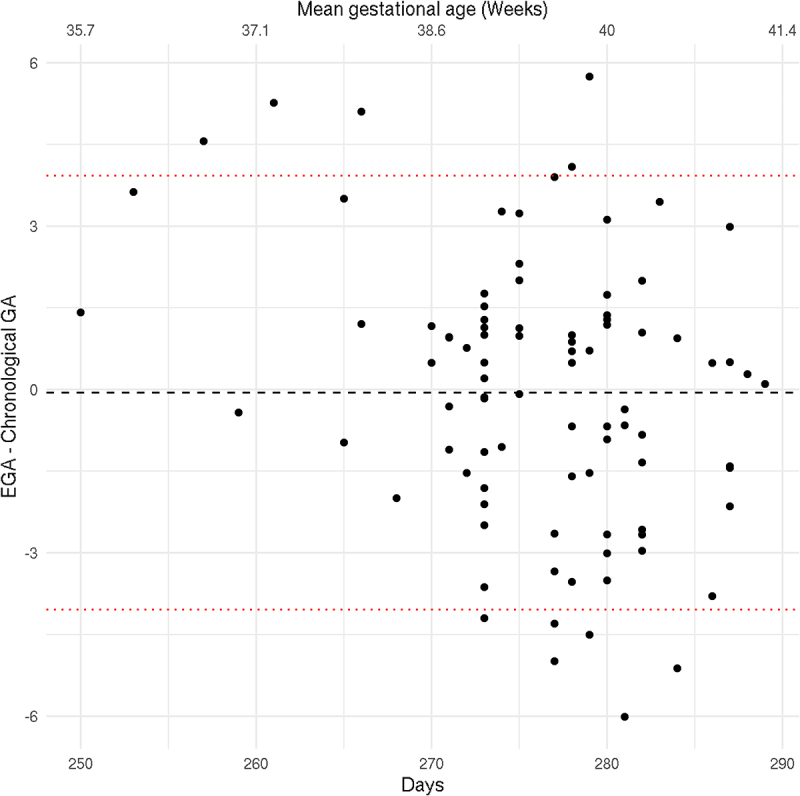
Bland-Altman plot displaying the difference in chronological gestational age (GA) and epigenetic gestational age (EGA) across the mean gestational age in days and weeks. To assess the agreement between chronological GA and EGA across pre-term, term and post-term GA, we applied the Bland-Altman approach below to our test subset. Red dotted line indicates the confidence interval from -4.04 to 3.93. The mean difference was calculated at -0.058 and displayed by black dotted line.-.

In the training set (*n* = 312), 2,049 CpG were FDR-significant and utilized to construct the multi-CpG site GA clock. The final elastic net penalized linear regression model included 233 CpGs; the regression parameter estimates for this model are included in Supplementary Table S4. We then applied the model to the test set (*n* = 78) and assessed for agreement between EGA and chronological GA. Model assessment metrics demonstrated agreement (R^2^ = 0.88, Pearson’s Correlation = 0.94, and Intraclass Correlation Coefficient = 0.94). Supplementary Table S3 displays the scatter plot of EGA and chronological GA. A Bland-Altman plot was also used to visualize the agreement between chronological GA and EGA (Figure 2). The mean difference was calculated at −0.058 days, with a 95% confidence interval (CI) = −4.04 – 3.94 days.

### Shared biological pathway enrichment between DNAm GA clocks

**Figure 3.**
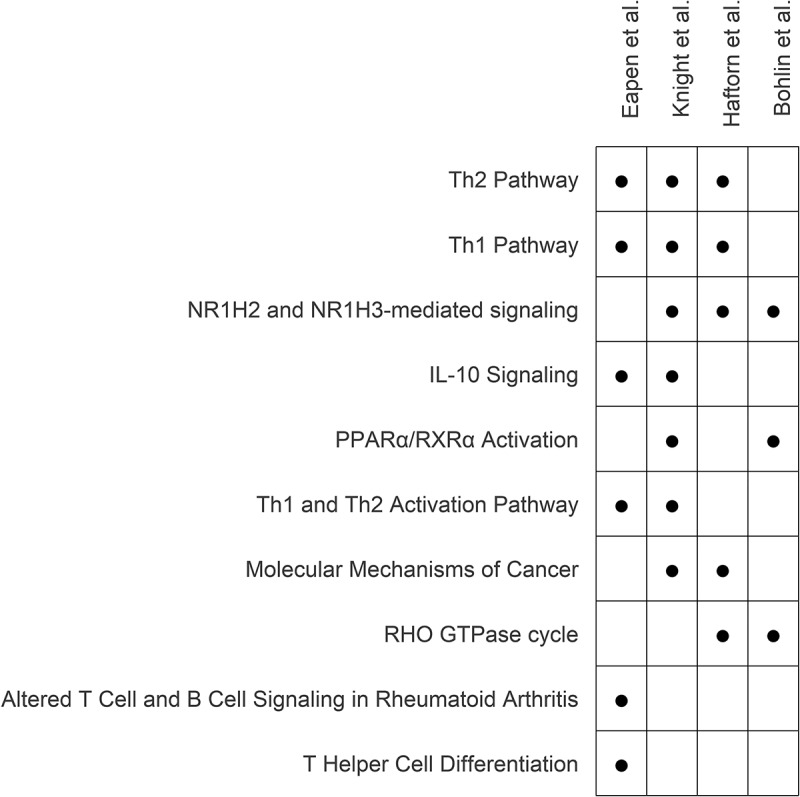
The top 10 biological pathways enriched across gestational age clocks. Pathway analyses via IPA was performed from the genes mapping to the CpGs within each of the four clocks, accounting for the differing background of genes represented on the respective arrays. For each pathway, enrichment *p*-values were combined across studies using the sum of the study specific pathway enrichment -log10 (*p*-values) and in this table, the pathways are displayed in their order of significance from top to bottom.

To compare biological pathways enriched between existing DNAm GA clocks and ours, we performed IPA on the genes mapping to the CpGs included in our clock (Supplementary Table S5) and four other EGA clocks (Haftorn, Supplementary Table S6; Knight, Supplementary Table S7; Bohlin, Supplementary Table S8) accounting for the differing backgrounds of genes represented on the respective arrays. The array used for each existing GA clock and number of CpG sites retained in eachare as follows: Bohlin et al. utilized the Illumina HumanMethylation450 [27] and retained 131 CpGs; Haftorn et al. utilized the Illumina MethylationEPIC 850K [28] and retained 176 CpGs, and Knight et al. utilized both the Illumina HumanMethylation27 Beadchip and Infinium HumanMethylation450 Beadchip [29] and retained 148 CpGs. Figure 3 displays the top 10 canonical biological pathways ranked based on the sum of the -log_10_p-values across the four GA clocks, and the full cross-clock pathway results are presented in Supplementary Table S9. The Knight clock had the most overlapping enriched pathways with the other clocks: ours (*n* = 4), Bohlin (*n* = 3), and Haftorn (*n* = 4). Although there was not a pathway shared by all clocks that met a nominal level of significance (*p* < 0.05), Th1, Th2, and NR1H2 and NR1H3-mediated pathways were enriched in three of the clocks. Of note, the Th1 and Th2 pathways were also captured in our pathway analysis based on all GA-associated CpG regions (Figure 1). Further, the Th1 and Th2 activation pathways enriched in that analysis (Figure 1) were also enriched in our clock CpGs and validated in the Knight clock (Figure 3).

Dot represents pathway enrichment *p*-value < 0.05. For all pathways in each study, detailed *p*-values are listed in Supplementary Table S5-S8.

### Environmental factors and gestational age

Next, individual prenatal/perinatal environmental exposures were evaluated for association between chronological GA, EGA, and EGAA in both single and multi-exposure models. The 22 exposures assessed are included in Table 3. In the single exposure models and after multiple test correction, delivery mode, baby birth weight z-score were the only exposures significantly (FDR adjusted *p*-value < 0.05) associated with chronological GA and EGA. When delivery mode was stratified by vaginal, planned, and unplanned C-section, planned C-section was significantly associated with a lower chronological GA (Coefficient [95% CI] = −4.38 [−7.17,-1.59], *p* = 0.002) and EGA (Coefficient [95% CI] = −4.56 [−7.15,-1.97], *p* = 0.001) while unplanned was associated with high chronological GA (Coefficient [95% CI] = 3.29 [0.39, 6.19], *p* = 0.026) and EGA (Coefficient [95% CI] = 2.89 [0.21,5.58], *p* = 0.035). Birth weight z-score was associated with higher chronological GA (Coefficient [95% CI] = 2.28 [1.16, 3.39], *p* < 0.001) and EGA (Coefficient [95% CI] = 2.10 [1.06, 3.14], *p* < 0.001). Although not meeting FDR significance, household income greater than $100,00 was associated with increased chronological GA (Coefficient [95%] = 5.79 [1.6, 9.99], *p* = 0.007) and EGA (Coefficient [95%] = 5.52 [1.63, 9.4], *p* = 0.006).

**Table 3.** Single prenatal and perinatal exposure associations with gestational age (GA) measures for each GA measure, unadjusted univariate linear regression models with single prenatal/perinatal environmental exposures were fit.

		Chronological gestational age			Epigenetic gestational age			Epigenetic gestational age acceleration		
Covariate	Levels	Coefficient	95% CI	*p-value	Coefficient	95% CI	*p-value	Coefficient	95% CI	*p-value
Maternal Age		−0.0037	(−0.21, 0.2)	0.972	−0.02	(−0.21, 0.17)	0.855	−0.01	(−0.06, 0.03)	0.547
Paternal Age		−0.12	(−0.28, 0.05)	0.161	−0.11	(−0.27, 0.04)	0.143	0.0035	(−0.03, 0.04)	0.853
BMI First Recorded in Pregnancy		−0.06	(−0.19, 0.08)	0.402	−0.06	(−0.18, 0.07)	0.355	−0.0013	(−0.03, 0.03)	0.933
BMI Last Recorded in Pregnancy		0.01	(−0.13, 0.15)	0.908	0.01	(−0.12, 0.13)	0.925	−0.002	(−0.03, 0.03)	0.900
Birth Weight z-score		2.28	(1.16, 3.39)	<0.001^‡^	2.10	(1.06, 3.14)	< 0.001^‡^	−0.18	(−0.43, 0.08)	0.172
Parity		−0.49	(−1.46, 0.48)	0.323	−0.36	(−1.26, 0.54)	0.429	0.12	(−0.09, 0.34)	0.257
Marital Status	Unmarried	Ref	Ref	Ref	Ref	Ref	Ref	Ref	Ref	Ref
	Married	1.88	(−0.43, 4.19)	0.111	1.90	(−0.24, 4.05)	0.082	0.03	(−0.49, 0.55)	0.914
Urban Residence	Suburban	Ref	Ref	Ref	Ref	Ref	Ref	Ref	Ref	Ref
	Urban (Detroit)	−0.86	(−3.06, 1.34)	0.443	−0.68	(−2.72, 1.36)	0.513	0.18	(−0.31, 0.67)	0.476
Household Income	<20k	Ref	Ref	Ref	Ref	Ref	Ref	Ref	Ref	Ref
	20k- <40k	2.27	(−1.56, 6.09)	0.245	1.92	(−1.62, 5.46)	0.288	−0.35	(−1.21, 0.51)	0.428
	40k- <80k	3.37	(−0.27, 7.01)	0.070	3.22	(−0.15, 6.6)	0.061	−0.14	(−0.96, 0.68)	0.730
	80k- <100k	1.53	(−2.73, 5.79)	0.480	0.65	(−3.3, 4.59)	0.747	−0.88	(−1.84, 0.07)	0.070
	≥100k	5.79	(1.6, 9.99)	0.007	5.52	(1.63, 9.4)	0.006	−0.27	(−1.22, 0.67)	0.567
	refuse to answer	1.35	(−2.95, 5.65)	0.539	1.13	(−2.85, 5.12)	0.577	−0.22	(−1.18, 0.75)	0.662
Maternal Education	<HS diploma	Ref	Ref	Ref	Ref	Ref	Ref	Ref	Ref	Ref
	HS diploma	−4.14	(−10.19, 1.9)	0.179	−3.23	(−8.86, 2.39)	0.259	0.91	(−0.45, 2.27)	0.191
	Some college	−5.25	(−10.8, 0.3)	0.064	−4.18	(−9.35, 0.98)	0.112	1.07	(−0.18, 2.32)	0.094
	≥Bachelor’s degree	−1.57	(−7.2, 4.06)	0.584	−0.96	(−6.2, 4.27)	0.718	0.61	(−0.66, 1.88)	0.347
Delivery Mode	Vaginal	Ref	Ref	Ref	Ref	Ref	Ref	Ref	Ref	Ref
	Planned C-section	−4.38	(−7.17, −1.59)	0.002‡	−4.56	(−7.15, −1.97)	0.001‡	−0.18	(−0.82, 0.45)	0.573
	Unplanned C-section	3.29	(0.39, 6.19)	0.026	2.89	(0.21, 5.58)	0.035	−0.40	(−1.06, 0.26)	0.236
First Born	No	Ref	Ref	Ref	Ref	Ref	Ref	Ref	Ref	Ref
	Yes	−0.30	(−2.59, 1.99)	0.797	−0.10	(−2.23, 2.03)	0.929	0.20	(−0.31, 0.72)	0.436
Number of Previous Live Births	0	Ref	Ref	Ref	Ref	Ref	Ref	Ref	Ref	Ref
	1	−0.13	(−3.32, 3.05)	0.935	−0.36	(−3.32, 2.59)	0.809	−0.23	(−0.95, 0.48)	0.525
	2	−1.21	(−4.34, 1.92)	0.447	−1.76	(−4.66, 1.15)	0.235	−0.55	(−1.25, 0.16)	0.127
	3	−2.89	(−6.58, 0.81)	0.126	−2.98	(−6.41, 0.45)	0.089	−0.09	(−0.92, 0.74)	0.832
	≥4	−3.99	(−7.65, −0.34)	0.032	−3.85	(−7.24, −0.45)	0.026	0.15	(−0.67, 0.97)	0.722
Infant Gender	Male	Ref	Ref	Ref	Ref	Ref	Ref	Ref	Ref	Ref
	Female	0.77	(−1.41, 2.95)	0.490	0.89	(−1.14, 2.91)	0.389	0.12	(−0.37, 0.61)	0.622
Maternal Antibiotic Use during Pregnancy	No	Ref	Ref	Ref	Ref	Ref	Ref	Ref	Ref	Ref
	Yes	−0.63	(−2.85, 1.59)	0.575	−0.25	(−2.31, 1.8)	0.810	0.38	(−0.12, 0.88)	0.139
Maternal Antifungal Use during Pregnancy	No	Ref	Ref	Ref	Ref	Ref	Ref	Ref	Ref	Ref
	Yes	−0.90	(−3.78, 1.99)	0.540	−1.13	(−3.8, 1.54)	0.407	−0.23	(−0.89, 0.43)	0.494
Environmental Tobacco Smoke	No	Ref	Ref	Ref	Ref	Ref	Ref	Ref	Ref	Ref
	Yes	−1.80	(−4.3, 0.7)	0.157	−1.70	(−4.02, 0.62)	0.150	0.10	(−0.46, 0.66)	0.726
Mom Smoking Status	No	Ref	Ref	Ref	Ref	Ref	Ref	Ref	Ref	Ref
	Yes	−2.31	(−5.75, 1.13)	0.188	−2.01	(−5.2, 1.19)	0.217	0.30	(−0.47, 1.07)	0.446
Indoor Pets	No	Ref	Ref	Ref	Ref	Ref	Ref	Ref	Ref	Ref
	Yes	0.73	(−1.52, 2.99)	0.523	0.42	(−1.67, 2.52)	0.692	−0.31	(−0.82, 0.19)	0.225
Outdoor Pets	No	Ref	Ref	Ref	Ref	Ref	Ref	Ref	Ref	Ref
	Yes	2.18	(−3.31, 7.68)	0.435	2.07	(−3.03, 7.17)	0.425	−0.11	(−1.34, 1.12)	0.855
Maternal Race	African American	Ref	Ref	Ref	Ref	Ref	Ref	Ref	Ref	Ref
	European American	1.24	(−1.37, 3.85)	0.351	1.14	(−1.29, 3.56)	0.357	−0.10	(−0.69, 0.48)	0.726
	Other	1.15	(−2.02, 4.32)	0.477	1.39	(−1.56, 4.34)	0.354	0.24	(−0.47, 0.95)	0.507
Infant’s Race	African American	Ref	Ref	Ref	Ref	Ref	Ref	Ref	Ref	Ref
	European American	1.39	(−1.22, 3.99)	0.296	1.23	(−1.19, 3.65)	0.317	−0.16	(−0.74, 0.43)	0.599
	Other	2.08	(−1.2, 5.37)	0.214	2.05	(−1, 5.1)	0.187	−0.03	(−0.77, 0.71)	0.936

Abbreviations: CI, confidence interval; C-section, Caesarean section; GA, gestational age; HS, high school; k, thousand; Ref, reference.

Coefficient: Regression parameter estimate for CpG site – gestational age model.

*F-test evaluating significant differences for continuous or dichotomous characteristics for each GA outcome.

‡FDR-adjusted *p*-value < 0.05.

To assess whether groups of environmental factors were associated with GA assessments, a multi-exposure model was constructed for each of the three GA measures using a backward selection procedure, and the resulting multi-exposure models are summarized in Table 4. Supplementary Table S10 presents the effect estimates for all exposures examined in the multi-exposure model. The prenatal/perinatal multi-exposure models explained more of the variation in chronological GA and EGA (adjusted R^2^ = 0.112 and 0.132, respectively) in comparison to EGAA (adjusted R^2^ = 0.012). Further, there was general agreement between multi-exposure models of both chronological GA and EGA, and the directions of effect also agreed with their single exposure estimates. Environmental factors retained in models for both chronological GA and EGA were first born status, birth weight Z-score, marital status, and delivery mode. Maternal smoking and paternal age were retained in the model for chronological GA but not EGA, while maternal BMI first and last recorded in pregnancy, infant race, and number of pregnancies were retained in the model for EGA but not chronological GA. In the EGA and chronological GA models, birth weight and mode of delivery were statistically significant (*p* < 0.001), and marital status was also statistically significant in the chronological GA model. In the multi-exposure model of EGAA, firstborn status (*p* = 0.035) and parity (*p* = 0.017) were retained and associated with increased EGAA. First born status was the sole exposure retained in all three measures of GA.

**Table 4.** Prenatal and perinatal multi-exposure models of chronologic gestational age, epigenetic gestational age, and epigenetic age acceleration. To account for the role of multiple environmental factors affecting gestational age (GA) measures, a multi-exposure model was constructed separately for each GA measure using a backward selection. In each, the model construction began with the inclusion of all 22 prenatal/perinatal factors, and the Akaike Information Criteria (AIC) was utilized to determine exposures that were retained.

		Chronological gestational age			Epigenetic gestational age			Epigenetic gestational age acceleration		
Covariate	Levels	Coefficient	95% CI	*p-value	Coefficient	95% CI	*p-value	Coefficient	95% CI	*p-value
First born	No	Ref	Ref	Ref	Ref	Ref	Ref	Ref	Ref	Ref
	Yes	−2.42	(−5.8, 0.96)	0.160	−2.71	(−6.04, 0.61)	0.109	0.87	(0.06, 1.68)	0.035
Parity		−1.29	(−2.71, 0.12)	0.073				0.42	(0.07, 0.76)	0.017
Birth Weight z-score		2.44	(1.3, 3.58)	<0.001	2.35	(1.26, 3.45)	<0.001			
Delivery Mode	Vaginal	Ref	Ref	Ref	Ref	Ref	Ref			
	Planned C-section	−6.13	(−9.12, −3.15)	<0.001	−5.94	(−8.7, −3.19)	<0.001			
	Unplanned C-section	3.24	(0.25, 6.24)	0.034	2.64	(−0.17, 5.46)	0.066			
Marital Status	Unmarried	Ref	Ref	Ref	Ref	Ref	Ref			
	Married	2.23	(−0.35, 4.81)	0.090	2.57	(0.17, 4.98)	0.036			
Paternal Age		−0.14	(−0.31, 0.03)	0.104						
Mom Smoking Status	No	Ref	Ref	Ref						
	Yes	−2.69	(−6.4, 1.01)	0.153						
BMI First Recorded in Pregnancy					−0.36	(−0.78, 0.06)	0.095			
BMI Last Recorded in Pregnancy					0.32	(−0.12, 0.77)	0.153			
Infant Race	African American				Ref	Ref	Ref			
	European American				−2.74	(−5.47, −0.01)	0.049			
	Other				0.11	(−3.26, 3.48)	0.947			
Number of Previous Live Births	0				Ref	Ref	Ref			
	1				−1.48	(−5.38, 2.42)	0.457			
	2				−3.51	(−7.61, 0.6)	0.094			
	3				−4.99	(−9.66, −0.33)	0.036			
	≥4				−6.97	(−11.81, −2.13)	0.005			

Note: The model multiple R2/adjusted R2 estimates for chronologic gestational age, epigenetic gestational age, and epigenetic age acceleration were 0.134/0.112, 0.167/0.132, and 0.018/0.012, respectively.

Abbreviations: CI, confidence interval; C-section, Caesarean section; GA, gestational age; HS, high school; k, thousand; Ref, reference; Coefficient, regression parameter estimate for CpG site – gestational age model.

*F-test evaluating significant differences for continuous or dichotomous characteristics for each GA outcome.

## Discussion

In our study of DNAm and GA, our region-based EWAS results identified genes in the Th1 and Th2 signaling, IL10 signaling, and macrophage classical activation pathways as being significantly enriched in DNAm associated with chronological GA. Additionally, CpG sites retained in our GA clock also highlight these Th1, Th2, and IL-10 signaling pathways. The early life immune system is noted to be associated with GA, with highlighted pathways involving pro- and anti-inflammatory immune mechanisms and variations in T cell populations [30].

In our single CpG site and region-based EWAS, HLA-class II genes (*HLA-DMA, HLA-DMB, HLA-DQB1, HLA-DQB2, and HLA-DRB1*) and *NFKB1* were the most significant genes implicated in pathways significantly enriched for chronological GA. Among the HLA-class II genes identified, expression of HLA-DRB1 has been previously reported to be associated with both age-related changes in brain structure and cognitive performance [31,32], as well as with longevity beyond 85 years of age. Further, single cell proteomic studies of cord blood have found associations with GA for both T-reg pathways and NFKB1 signaling in antigen presenting cells expressing HLA-class II genes [30], and T-cell pathway skewing and chronic antigen stimulation have been previously noted to mediate immunosenescence [33]. Although all components of the innate and adaptive immune system are adversely affected to varying extents by aging, antigen presentation, T cell activation, and NFKB1 signaling appear to be particularly sensitive and important to the aging process [34]. Our findings highlight the role of early life DNAm at HLA-class II genes and *NFKB1* that may be regulating these immune pathways through epigenetic gene regulation.

We herein present a GA clock based on the Asthma&Allergy array (Pearson Correlation *r* = 0.94, R^2^ = 0.88). As DNAm arrays have evolved, the accuracy of GA prediction has also changed. Our clock had higher model fit when compared to the Bohlin clock (R^2^ = 0.66) and the recently developed Haftorn clock (R^2^ = 0.71) [28]. This variation between chronological GA and predicted EGA is likely due to the differences in both the number and epigenome-wide coverage of CpGs associated with GA. However, our findings suggest that the accuracy of our DNAm-based GA clock is comparable to other similar existing methods, particularly between 35 and 40 weeks.

When comparing previously published GA clocks to ours based on enriched pathways, there were multiple findings of note. First, many of the immune related pathways (Th1, Th2, and IL-10 signaling) captured in our pathway analyses based on 1) all GA-associated region CpGs and 2) those CpGs in our clock were found to be enriched in at least one of the other three clocks. These findings not only validate the importance of immune-related pathways in early life aging, which is consistent with the effects demonstrated in the broader aging literature [35], but also reveal that these immune pathways are important in the prediction of GA. Second, these analyses highlighted NR1H2 and NR1H3 mediated signaling across three of the four clocks. NR1H2 and NR1H3 are hepatic nuclear receptors that have been associated with lipogenesis [36] and tumor cell growth [36,37]. Furthermore, studies have found associations of accelerated epigenetic aging with obesity [38] and incidence across multiple tumor types [39]. These studies with ours strengthens the association of epigenetic aging and immunomodulation, with potential links to disease.

The environment is noted to have effects on DNAm, and consistently, our findings support the impact of multiple prenatal/perinatal factors on GA and EGAA. Previous studies investigating the association between prenatal environmental factors and DNAm with GA have primarily focused on maternal conditions (e.g., pre-eclampsia [40], maternal obesity [41]), prenatal medications [42], and smoking/pollutants [14]. Our study has taken a more agnostic approach, investigating a longer list of factors.

While maternal smoking has been associated with placental DNAm and decreased GA [43], our findings did not find a significant association of maternal smoking with chronological GA, EGA, or EGAA. SES, specifically low SES, has been associated with decreased GA [44]. Our findings show household incomes greater than $100,000 associated with increased chronological GA and EGA. Birth weight was associated with both chronological GA and EGA but not EGAA. The association between birth weight and EGA in our study may be more due to chronological GA than to biological age. Further work is needed, as previous studies have found that birthweight is associated with EGAA through childhood and into adulthood [45,46].

Our analyses of environmental variables also emphasize the impact of multiple prenatal and perinatal environmental factors in chronological GA, EGA, and EGAA. Specifically, first born status was retained in association with chronological GA, and EGAA. Parity has shown a correlation with chronological GA and preterm births [47], and also identified as a significant exposure in the Knight and Bohlin clock [48]. Previous studies have reported the effects of birth order on DNAm, with one candidate gene study reporting DNAm of genes in T-cell pathways to be associated with birth order [49,50]. One potential explanation for how birth order and parity are associated with these pathways could involve how the pregnancy process and placentation change with each subsequent pregnancy [49]. Lastly, infant’s race and maternal marital status were associated with EGA, potentially identifying SES factors that influence prenatal development. Further studies are needed to identify mechanisms behind these pathways that may have effects on biological aging and potential future health outcomes.

There are several strengths to our study. These include, but are not limited to, early DNAm assessment across preterm, term, and post-term GA; a large, diverse cohort (in terms of race and SES); and detailed prenatal/perinatal environmental exposure data. However, limitations to our study also exist. One such limitation is that our characterization of C-section as planned versus unplanned was chart abstracted and defined as whether the C-section was scheduled or not. Although most of the unplanned C-sections were likely laboring, there may be a small portion that were not (e.g., maternal preeclampsia, large-for-gestational age fetus). Future studies further stratifying delivery mode by labor status would help clarify the effects of labor on EGA and EGAA. Additionally, we did not have cell type composition available for our samples, which has been reported to contribute to DNAm variability in general [51] as well as DNAm associations with GA, particularly in nucleated red blood cells [52]. We therefore utilized CorrConf to indirectly estimate biological confounding factors, including cell composition [18]. Future studies of cord DNAm would have cell composition available to better account for this variation. While we did implement a rigorous training/testing approach for our EGA clock development and evaluation, we did not have an independent cohort to validate our findings. While this is a limitation, there was overlap between the pathways with enriched GA associated CpG included in our EGA model in comparison to other existing EGA clocks, which is evidence of consistency and validation. Also, in terms of our pathway analyses, given the differing number of CpGs that map to each gene represented on the array, we may be under-calling potentially significant GA-enriched pathways (i.e., truly GA associated genes not identified by our analysis due to lower coverage on the Asthma&Allergy array). However, there is no indication that the differential CpG coverage for genes on the array would result in biased identification of enriched pathways where no such enrichment exists. Further, while we evaluated multiple exposure associations with GA measures, the list was not exhaustive. Given our suggestive findings, future studies should expand the list of exposures investigated, including prenatal environmental exposures such as indoor/outdoor environmental pollution measures which have been shown to impact DNAm [53,54].

In conclusion, our findings highlight immune pathways and gene associated with cord-blood EGA. Our findings additionally show influence of prenatal/perinatal factors on chronological GA, EGA, and EGAA, which may have an influence on subsequent biological pathways throughout life. Future studies applying EGA clocks to disease outcomes would benefit from incorporating the influence of prenatal/perinatal environmental factors to identify mechanisms in risk of disease.

## Supplementary Material

Supplemental Material

Supplementary Figure 1 revision 2.png

Supplementary Figure 3 Scatter plot of EGA versus chronological age in test set revision 2 final.png

Supplementary Figure 2 QQ plot final revision 2.png

## Data sharing

Datasets analyzed in this study are not publicly available; consent for public release of epigenetic data from participants was not obtained from participants. However, data to generate figures and tables are available from the corresponding author with the appropriate permission from the WHEALS study team and investigators upon reasonable request and Institutional Review Board approval.

## Abbreviations

DNAm: DNA methylation
GA: Gestational age
EGA: Epigenetic gestational age
EGAA: Epigenetic gestational age acceleration
EWAS: Epigenome-wide association study
Th1: T helper 1
Th2: T helper 2
WHEALS: Wayne County Health, Environment, Allergy and Asthma Longitudinal Study
