# Supplementary figures and images for: Immune pathways and prenatal/perinatal environmental exposures contribute to epigenetic gestational age prediction and acceleration
Source: Epigenetics. 2026 Jan 20;21(1):2610521. doi: 10.1080/15592294.2025.2610521 (PMC12826722; doi:10.1080/15592294.2025.2610521)

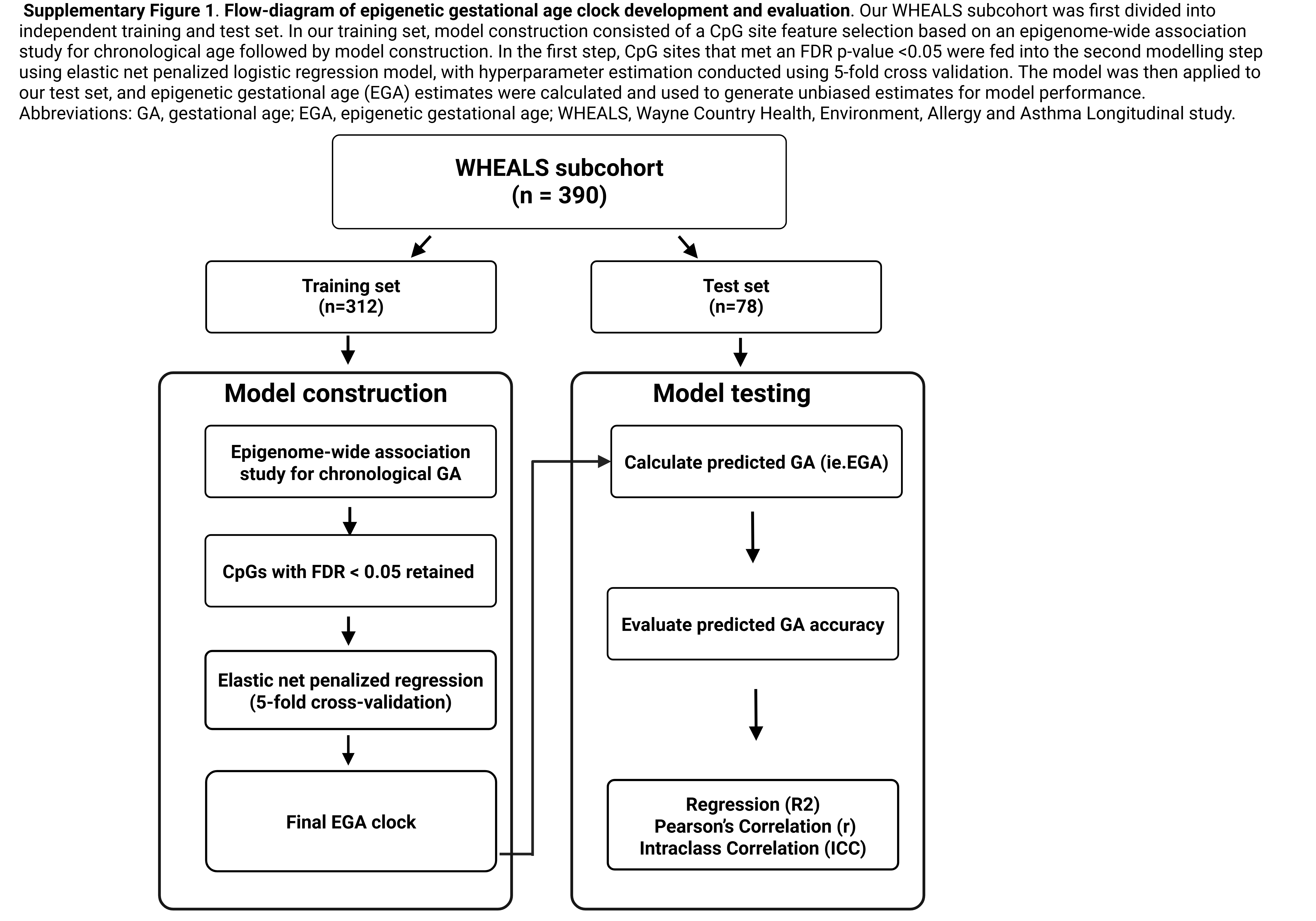

Supplement: Supplementary Figure 1 revision 2.png [file KEPI_A_2610521_SM4357.png]

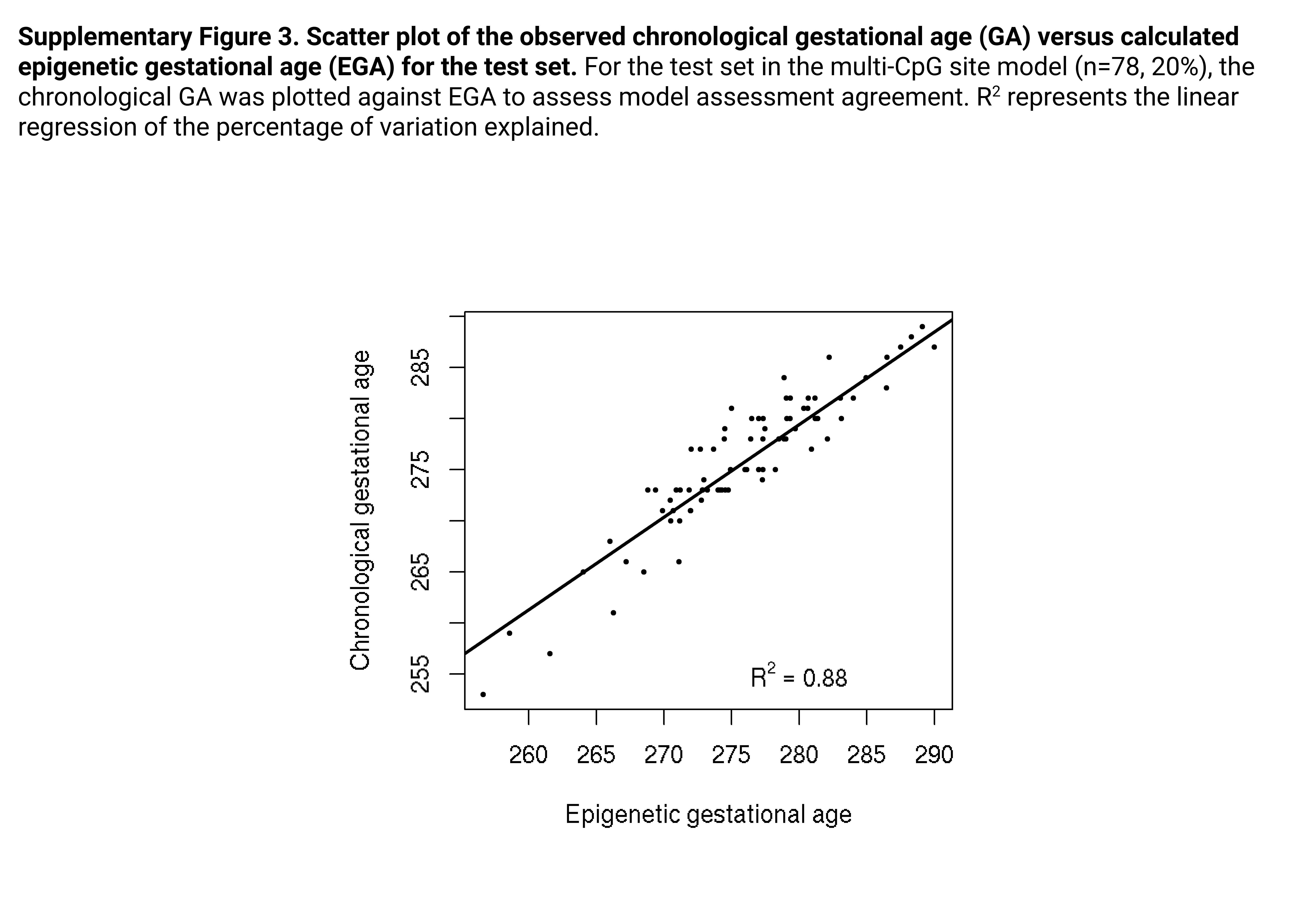

Supplement: Supplementary Figure 3 Scatter plot of EGA versus chronological age in test set revision 2 final.png [file KEPI_A_2610521_SM4347.png]

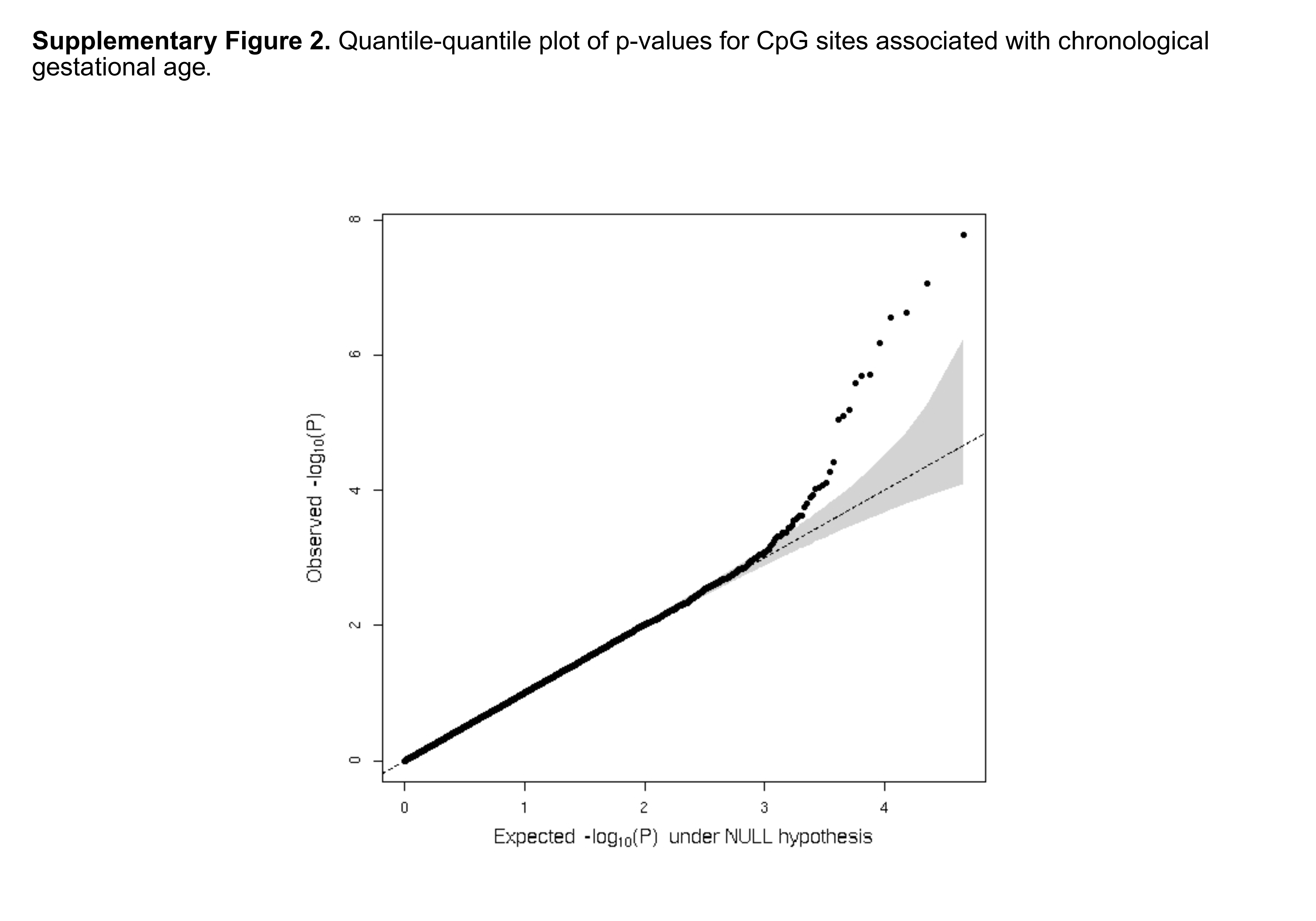

Supplement: Supplementary Figure 2 QQ plot final revision 2.png [file KEPI_A_2610521_SM4345.png]
